# Beliefs about causes of weight gain, effective weight gain prevention strategies, and barriers to weight management in the Australian population

**DOI:** 10.1080/21642850.2013.872036

**Published:** 2014-01-21

**Authors:** Rachel Dryer, Nicole Ware

**Affiliations:** ^a^ School of Psychology, Charles Sturt University, Bathurst, NSW2795, Australia

**Keywords:** weight gain, obesity, beliefs, weight management, general public

## Abstract

*Purpose:* To identify beliefs held by the general public regarding causes of weight gain, weight prevention strategies, and barriers to weight management; and to examine whether such beliefs predict the actual body mass of participants. *Methods:* A questionnaire-based survey was administered to participants recruited from regional and metropolitan areas of Australia. This questionnaire obtained demographic information, height, weight; as well as beliefs about causes of weight gain, weight prevention strategies, and barriers to weight management. *Results:* The sample consisted of 376 participants (94 males, 282 females) between the ages of 18 years and 88 years (mean age = 43.25, SD = 13.64). The range and nature of the belief dimensions identified suggest that the Australian public have an understanding of the interaction between internal and external factors that impact on weight gain but also prevent successful weight management. Beliefs about prevention strategies and barriers to effective weight management were found to predict the participants’ actual body mass, even after controlling for demographic characteristics. *Conclusions:* The general public have a good understanding of the multiple contributing factors to weight gain and successful weight management. However, this understanding may not necessarily lead to individuals adopting the required lifestyle changes that result in achievement or maintenance of healthy weight levels.

Conditions of being overweight and obesity have long been associated with enormous psychological, health and economic costs on individuals, families and communities (Stroebe, 2008). However, more recently, these costs have been reported to occur with any level of weight gain. Increases in body weight of less than 5 kg have been found to be associated with increased disease load, with the associations occurring even within the healthy body mass index (BMI) range (Lim, Norman, Clifton, & Noakes, 2008). Biological indicators of health risk (e.g. diabetes, cardiovascular diseases) have also been shown to have a strong monotonic relationship with body weight from the lowest BMIs to the highest BMIs, with higher body weight associated with greater health risk (Zajacova, Dowd, & Burgard, 2011). Therefore, it is not surprising that the prevention of weight gain has become a focus of public discussion and debate, with numerous theories of causes and solutions in the public arena (Faith, Fontaine, Baskin, & Allison, 2007; National Obesity Task Force Obesity Working Group, 2009).

In Australia, the prevalence rates of being overweight and obesity are amongst the highest in the world (Thorburn, 2005). Between 2011 and 2012, approximately 60% of the adult population were classified as either overweight or obese, with over 25% of these individuals meeting the criteria for obesity (Australian Bureau of Statistics, 2012). Note that being overweight is often defined as having a BMI of 25–30, while obesity is defined as having a BMI of 30 or higher. In an attempt to stem this rapidly increasing health problem, the Australian National Health and Medical Research Council (2013) has released clinical guidelines that advocate supporting and encouraging individuals to change their lifestyle and behaviour to routinely include healthy eating habits and increased physical activity. The aim of these guidelines are to prevent initial weight gain in normal weight adults, as well as preventing further weight gain in those who are already overweight or obese. However, for population-level interventions to be effective, they need to be accepted and supported by the general population. This entails an understanding of the beliefs and attitudes held within the community (Lombard, Deeks, & Teede, 2009).

Various studies have examined the beliefs about obesity amongst both lay and professional groups. These studies have generally found that while most people recognise that multiple factors contribute to obesity, both lay and professional groups are more likely to place more importance on causes that are within the individual's control (e.g. not enough exercise, eating too much and too many unhealthy foods), and provide relatively weaker endorsement for social and biological causes (e.g. lack of education, low income, genetics, and hormones) (Harvey, Summerbell, Kirk, & Hills, 2002; Ogden & Flanagan, 2008; Okonkwo & While, 2010). Furthermore, while similar causal beliefs are held for both obesity and being overweight, those who are obese are often seen by professionals as being more responsible for their weight than those who are overweight (Harvey et al., 2002).

Recently, McFerran and Mukhopadyay (2013) conducted a series of studies to examine whether causal beliefs about obesity predicted participants' actual BMI. They reported that individuals primarily believed obesity to be caused by either a lack of exercise or a poor diet. Individuals who believed obesity to be primarily caused by a lack of exercise were more likely to be overweight and have higher BMIs than those who held a diet explanation of obesity. This relationship was found even after controlling for the demographic characteristics of participants as well as other known correlates of BMI. The researchers further examined this relationship in an experiment that involved presenting participants with a passage containing either a ‘diet’ or an ‘exercise’ explanation of obesity. Those who were presented with the ‘exercise’ explanation were found to consume more food than those participants who were presented with a ‘diet’ explanation. The authors concluded that causal beliefs of obesity have pervasive and systematic influences on the individuals' BMI and food consumption.

While many of the belief studies on obesity have primarily focused on causal explanations, a few have also looked at the beliefs about solutions to obesity. These studies have found that there is community and professional support for a range of intervention strategies, but these strategies are not seen to be equally effective. For example, Ogden and Flanagan (2008) found that general medical practitioners and lay individuals provided the strongest endorsement for the use of support groups, while the use of medication was the least accepted solution for both groups. This study also found that both general practitioners and the general public were ambivalent about the effectiveness of other intervention strategies involving surgery, counselling, and policy changes. In contrast, Okonkwo and While (2010) found that the most endorsed interventions amongst university students where those involving dietary (e.g. eating more fruits and vegetables, eating smaller portion sizes) and behavioural (e.g. exercising and reducing sedentary activities) changes. There was also widespread support for government actions such as restrictions on food advertisements targeted at children, banning of unhealthy food in schools, and mandated food labelling at restaurants and takeaway outlets as solutions to obesity. Okonkwo and While's study is also one of the few studies that have examined possible barriers to weight management. They found that many of their participants believed that the key barriers to adopting weight management strategies were individual motivation and environmental factors (e.g. busy lifestyle, cost of weight management strategies).

These studies provide insight into beliefs about obesity. However, the methodologies used to explore beliefs have involved either a limited number of predetermined questions or the use of predetermined categories to code participants' responses to open-ended questions, thereby limiting the ability of these studies to explore fully the range of beliefs and attitudes about obesity. Furthermore, obesity is a recognised medical condition that refers to an excess of body fat (Ogden, Carroll, & Legal, 2003). Obesity has also often been conceptualised as a biological deviation from the ‘normal’ healthy state (Jutel, 2006). In contrast weight gain is less visibly recognisable, is susceptible to fluctuations over the lifespan and affects a larger proportion of the population. Individuals may also fail to recognise their own weight gain over time and fail to recognise their own weight problems (Ziebland, Thorogood, Fuller, & Muir, 1996). Consequently, beliefs and attitudes about weight gain may be different to those of obesity.

One of the few studies that have examined the issue of weight gain was conducted by Jackson, Ball, and Crawford (2001), who examined the beliefs about the causes of personal weight gain and loss amongst the general community. They found that many of the participants did not attribute their weight gain purely to changes in their physical activity level or diet. Instead, they attributed the weight gain to other unspecified causes. Despite the impressive sample size, this study had methodological limitations similar to the aforementioned studies on obesity with regards to exploring the full range of beliefs/attitudes surrounding weight gain.

In consideration of some of these important questions that had not been adequately addressed in previous research, the current study had two primary goals. First, it sought to conduct a more comprehensive examination of the range of beliefs/attitudes about weight gain in adults by addressing the methodological limitations of previous studies. Numerous questionnaire items were generated based on pertinent information from both the general community and the literature on obesity and weight gain. Unlike many of the previous studies that have primarily focused on causal explanations, this study also examined beliefs about solutions and barriers to weight gain. Second, the current study examined whether McFerran and Mukhopadyay's (2013) findings could be replicated using a more comprehensive approach to exploring beliefs. Therefore, this study examined whether beliefs about weight gain (i.e. cause, solutions, and barriers) predicted the participants' BMI.

## Method

### Participants

The 376 participants consisted of adult men (*N* = 94) and women (*N* = 282) between the ages of 18 and 88 years (mean age = 43.25, SD = 13.64). These participants were recruited from regional and metropolitan areas of Australia. Postcode and suburb information were used to classify participants according to socioeconomic status (SES; using the Australian Bureau of Statistics Socioeconomic Indexes for Areas [SEIFA], 2006) and geographical location (using the Australian Bureau of Statistics Standard Geographical Areas – Remoteness Structure). A large proportion of the participants (77.13%) fell within the middle to high SES, with only 22% of the participants falling in the low SES range. The majority of the participants (65.16%) were located in inner-regional areas of Australia, with smaller proportions located in major cities (15.16%) and in outer-regional/very remote locations (19.15%). Using international BMI reference values (World Health Organization [WHO], 2006), it was determined from information provided by participants that over half of the participants (i.e. 67.2%) were overweight or obese. A large proportion of the participants (68%) indicated that they had gained weight, with only 21.7% reporting that they had successfully returned to their usual body weight. Many of these participants (79.7%) reported that they were actively trying to lose or maintain their usual weight.

### Questionnaire

The questionnaire items were developed from both a pilot study and review of the pertinent literature. The pilot study involved semi-structured interviews with 20 participants (9 males, 11 females) between the ages of 18 and 74 years (mean age = 38.00, SD, 14.51) from both regional and metropolitan areas. These participants were not involved in the main study. Each participant was asked to provide possible causes of weight gain and barriers to effective weight management in adults. Any belief or explanation identified by two or more participants were phrased into a questionnaire item and included in the final questionnaire. The resultant items were supplemented by items drawn from the literature including government publications and policy documents (e.g. National Preventative Health Taskforce, 2010; National Obesity Task Force, 2006; National Obesity Task Force Obesity Working Group, 2009; Smith et al., 2005; WHO, 2000, 2002); previous studies that have explored beliefs about weight management, weight gain, and obesity (e.g. Ogden & Flanagan, 2008; Okonkwo & While, 2010); and current literature that examine scientific theories of weight gain and obesity (e.g. Eby & Colditz, 2008; Faith et al., 2007; Greener, Douglas, & van Teijlingen, 2010; Lombard et al., 2009; Stroebe, 2008; Swinburn & Egger, 2004; Swinburn, Caterson, Seidell, & James, 2004).

The final questionnaire consisted of of (i) 42 causal explanations of weight gain, (ii) 35 item on weight prevention strategies, and (iii) 34 items concerning barriers to weight management. Participants were asked to rate the importance of each item on a six-point scale (not at all important to extremely important). Participants were also requested to provide demographic information pertaining to gender, age, geographical location, education level, and current weight and height. Information about the participants' personal weight management history was also obtained.

### Procedure

Permission to undertake the study was obtained from an appropriately constituted Ethics Committee at Charles Sturt University (Bathurst, NSW). Participants were recruited through a snowballing sampling approach and random distribution of the questionnaire in shopping areas in New South Wales. All participants were provided with reply-paid or sealable envelopes to facilitate confidentiality and return of the questionnaires. Questionnaires were distributed over a three-week period across a variety of days and times in an effort to recruit participants from a variety of backgrounds. The questionnaire took approximately 15–20 min to complete. No inducements were provided to any of participants and return of the questionnaire was taken as provision of informed consent.

### Results

Separate principal component analyses were conducted on the participants' ratings of the (i) causal explanations, (ii) prevention strategies, and (iii) barriers to weight management. All item means were initially inspected for very low mean scores (i.e. mean scores of 1.0 or lower). None of the items met this criteria indicating that all items were seen as realistic causal explanations or solutions to weight management. Velicer's minimum average partial tests (Zwick & Velicer, 1986) were used to determine the appropriate number of components to extract. Varimax rotation was used to derive orthogonal dimensions to simplify interpretation. Items were regarded as contributing to the interpretation of a component only if their loadings were >.30 on the primary component and no more than .20 on any other component. Mean component scores were calculated using a sum of scores by component divided by the number of items (DiStefano, Zhu, & Mĭndrilă, 2009).

#### Causal beliefs of weight gain

A five component solution, which explained 50.57% of the variance, was extracted from the ratings of the 42 items. Fifteen items had similar loadings on two or more of the components and were therefore excluded from further analyses (see Table 1). The first component, labelled *self-control*, accounted for 12.27% of the variance and consisted of eight items relating to a lack of control over diet and exercise. The second component, labelled *lifestyle limitations* (11.37% of the variance), consisted of five items related to the higher cost of healthy eating, low prices of high fat/high sugar food, and the influence of long and irregular work hours. The items that loaded highly on the third component (10.63% of the variance) largely reflected psychological vulnerabilities (e.g. depression, stress, and low self-esteem). Accordingly, this component was labelled *psychological*. The fourth component, labelled *biological/medical* (8.27% of the variance), consisted of four items related to hormonal, metabolic, and medication-related causes. The final component, explaining 8.10% of the variance, was labelled *modern living* and consisted of four items reflecting the reduction in physical activity through the use of cars, modern appliances, and electronic entertainment as well as consuming too many ‘diet’ foods.

**Table 1.** Rotated component loadings, the mean rating (and standard deviation), and ranking for each causal belief item.

Causal belief components and items	Mean	SD	Rank	1	2	3	4	5
*Self-control* (Cronbach's *α* = .81)	4.22	0.63						
Eating the wrong types of foods	4.50	0.86	2	0.68				
Eating more food than you need	4.48	0.87	1	0.64				
Not enough physical activity/exercise	4.41	0.91	3	0.62				
Lack of self-control	4.09	1.07	6	0.60				
Eating too many convenience foods/takeaways	4.12	1.10	5	0.58				
Enjoying high fat/high sugar ‘bad’ foods	4.30	1.01	4	0.55				
Too much snacking	3.84	1.05	8	0.54				
Being lazy	3.96	1.20	7	0.54				
*Lifestyle limitations* (Cronbach's *α* = .76)	3.12	0.98						
Lack of awareness of problems with current eating/exercise habits	3.12	1.29	20		0.63			
Working long hours	3.15	1.40	19		0.58			
Low price of high fat/high sugar foods compared with fruit and vegetables			16		0.57			
Shift work/irregular working hours	2.92	1.43	25		0.56			
High cost of healthy foods (e.g. fruits, vegetables, grains, lean meat)	3.16	1.41	18		0.51			
*Psychological* (Cronbach's *α* = .80)	3.21	1.00						
Poor self-confidence	2.94	1.31	24			0.68		
Loneliness/social isolation	3.32	1.39	14			0.60		
Low self-esteem	3.30	1.31	13			0.59		
Depression	3.39	1.39	12			0.58		
Stress	3.46	1.28	11			0.57		
Normal part of growing old (i.e. aging)	2.80	1.25	26			0.53		
*Biological/medical* (Cronbach's *α* = .80)	3.12	1.08						
Medical conditions (e.g. thyroid problem)	3.25	1.43	15				0.75	
Side effects of medication	3.05	1.38	22				0.73	
Hormonal/pregnancy-related changes in metabolism	3.18	1.39	17				0.70	
Slow metabolism	2.99	1.29	23				0.60	
*Modern living* (Cronbach's *α* = .72)	3.19	0.95						
Increased use of modern appliances rather than manual labour (e.g. ride-on mowers, remote controls)	3.10	1.31	21					0.69
Increased use of cars over walking/cycling	3.49	1.20	10					0.68
Increased participation in sedentary leisure activities (e.g. TV, computers, electronic games)	3.68	1.21	9					0.64
Eating too much of ‘diet’, ‘low fat,’ ‘fat free’ foods	2.39	1.38	27					0.43
The 15 items excluded from further analyses due to similar loadings on two or more components include:								
Emotional ‘comfort’ eating	Too much soft/fizzy drinks					Too much alcohol		
Larger portion sizes	Increased consumption of refined/processed foods					A lack of nutritional knowledge		
Poor family eating habits	Confusing other cues with hunger (e.g. boredom, thirst)					Disruptive life-events (e.g. divorce, grief)		
Genetic factors			Giving up smoking			Lack of time for meal planning		
Lack of physical activity at work			Advertising and marketing of unhealthy foods			Eating too little of ‘diet’, ‘low fat’, ‘fat free’ foods		

Note: This table also shows the reliability estimates for the five components.

A one-way repeated measures analysis of variance showed that there were significant differences in the overall endorsement for the five components (*F*(3.84, 1368.06) = 178, *p* < .0001, partial *η* ^2^ = .34).^1^ As can be seen from Table 1, the most endorsed causal component was *self-control*, with its eight items being in the top eight ranked items based on means. Pair-wise comparisons (with a Bonferroni corrected significance level of .005) confirmed that *self-control* was rated as significantly more important compared to the other four components (all *p* < .0001). The level of endorsement for the remaining four components did not differ from each other (all *p* > .005).

#### Beliefs about prevention strategies against weight gain

A five component solution which accounted for 54.45% of the variance was extracted from the data examining beliefs about the prevention strategies against weight gain. The first component, which accounted for 14% of the variance, was labelled *access to education/exercise* to reflect the six items comprising this component. The second component, labelled *healthier eating*, consisted of seven items related to improved diet and eating practices (11.25% of the variance). The third component consisted of five items related to increasing physical exercise and activity. This component, labelled *physical exercise/activity*, accounted for 10.40% of the variance. The three items comprising the fourth component, labelled *medication/dietary supplements* (9.78% of the variance), were concerned with taking medication or dietary supplements to prevent weight gain. The final component was labelled *reduced serving size* and consisted of three items concerned with reducing the serving size of convenience foods, snacks, and restaurant meals. This component accounted for 9.00% of the variance (see Table 2).

**Table 2.** Rotated component loadings, the mean rating (and standard deviation), and ranking for each item on prevention strategies against weight gain.

Prevention strategies	Mean	SD	Rank	1	2	3	4	5
*Access to education/exercise* (Cronbach's *α* = .87)	3.64	0.92						
Increased advertising of health information	3.55	1.16	15	0.76				
More affordable access to nutritionists and dieticians	3.59	1.22	13	0.74				
Increased education on food and nutrition	3.81	1.12	11	0.70				
Local government initiatives to increase access to inexpensive exercise areas and programmes	3.60	1.25	12	0.66				
Increased levels of health education regarding effects of weight gain	3.56	1.18	14	0.64				
Subsidy for gyms/trainers to lower costs	3.53	1.38	16	0.63				
*Healthier eating* (Cronbach's *α* = .78)	3.98	0.69						
Eating less high sugar food	4.13	0.94	7		0.66			
Increased availability of healthy foods	4.08	1.05	8		0.63			
Stress management	3.48	1.21	18		0.60			
Eating less fat	4.03	1.13	9		0.59			
Eating more healthy foods (e.g. fruits, vegetables, grains, lean meat)	4.65	0.63	1		0.57			
Meal planning	3.91	1.00	10		0.57			
Being more aware of what one is eating (e.g. Counting kilojoules)	3.49	1.28	17		0.38			
*Physical activity* (Cronbach's *α* = .79)	4.44	0.61						
Increased participation in physical activity/exercise	4.55	0.71	3			0.84		
Higher levels of physical activity	4.39	0.86	5			0.72		
Eating a balanced diet	4.60	0.71	2			0.64		
Lifestyle change to include regular healthy eating and physical activity	4.44	0.84	4			0.63		
Encourage the use of active forms of transport (e.g. walking, cycling)	4.17	0.99	6			0.58		
*Medication/dietary supplements* (Cronbach's *α* = .70)	2.29	1.08						
Use of dietary supplements (e.g. vitamins, fish oil)	2.33	1.39	23				0.67	
Use of medication	2.42	1.37	22				0.65	
Use of meal replacements (e.g. protein bars, shakes)	2.07	1.39	24				0.60	
*Reduced serving sizes* (Cronbach's *α* = .84)	3.18	1.20						
Reduced serving sizes of meals in restaurants	3.04	1.41	21					0.85
Smaller serving sizes of pre-packaged foods/takeaways	3.35	1.42	19					0.84
Reduced serving sizes of snacks	3.11	1.39	20					0.69
Eleven items excluded from further analyses due to similar loadings on two or more components include:								
Increased development of safe areas for physical activity (e.g. bicycle paths, parks)		Reduced serving sizes of meals at home						
Clear labelling of nutritional content of all foods		Engaging in non-food related social activities						
Counselling for emotional issues		Higher taxes on high fat and high joule ‘junk’ food making them more expensive						
Subsidies to lower cost of health foods (e.g. fruit, vegetables, grains, lean meat)		Limiting advertising of unhealthy foods						
Return to eating natural foods		Higher taxes on takeaway foods						
Support groups								

Note: The reliability estimates for the five components are also shown.

There were significant differences in the endorsement of these five prevention components (*F*(3.16, 1132.80) = 431.57, *p* < .0001, *η* ^2^ = .55).^2^ The *physical activity* component received the strongest endorsement as the most important way of preventing weight gain. The next most endorsed was the *healthier eating* component, followed by the *access to education/exercise* and the *reduced serving size* components. The *medication/dietary supplement* component received the least endorsement, with a mean rating below the midpoint of the scale. The mean rating for all five components was found to be significantly different from each other (*p* < .0001 in all cases).

#### Beliefs about the barriers to effective weight management

A four component solution, accounting for 55.93% of the variance, was extracted from the data examining barriers to effective weight management (see Table 3). The first component, which was labelled *limited resources/access*, accounted for 24.29% of the variance and consisted of 14 items. The majority of these items related to the high cost of healthy food, sports, and exercise facilities. Some of these items also reflected difficulties accessing health services and exercise facilities due to distance or safety concerns. The second component, labelled *nutritional knowledge* (12.39% of the variance), included items related to lack of nutritional knowledge and education, and cultural/family values about food and body weight. The five items comprising the third component were concerned with psychological vulnerabilities (i.e. depression, lack of self-esteem, poor self-confidence) and biological predispositions (i.e. genetics, slow metabolism). Accordingly, this component was labelled *biological & psychological vulnerabilities* (11.82% of the variance). The final component was labelled *self-control & motivation* (7.43% of the variance) as it consisted of three items related to laziness, lack of self-control, and motivation.

**Table 3.** Rotated component loadings, the mean rating (and standard deviation), and ranking for each item relating to barriers to weight management.

Barriers to weight management components and items	Mean	SD	Rank	1	2	3	4
*Limited resource/access* (Cronbach's *α* = .92)	3.37	0.95					
Cost of sporting activities	3.13	1.39	19	.79			
Cost of physical activities (e.g. gym membership)	3.58	1.41	6	.79			
Cost of active leisure activities	3.20	1.35	15	.79			
Cost of weight management services (e.g. dieticians)	3.26	1.36	13	.73			
Cost of health foods (e.g. fruits, vegetables, grains, lean meat)	3.55	1.33	8	.72			
Limited resources (e.g. time, money).	3.57	1.35	7	.70			
Long distance between services/facilities making the use of cars necessary	3.19	1.40	17	.68			
Lack of safe areas for exercise	2.95	1.38	23	.65			
Difficulty accessing health services	2.86	1.40	24	.61			
Ease and convenience of unhealthy options (e.g. drive the car, eat takeaway food)	3.77	1.78	4	.52			
The health benefits of maintaining ideal weight are long-term making it difficult to maintain motivation	3.49	1.21	9	.43			
Lack of time for planned exercise	3.65	1.28	5	.35			
*Nutritional knowledge* (Cronbach's *α* = .82)	3.18	1.07					
Lack of nutritional knowledge	3.29	1.25	12		.77		
Lack of nutritional education	3.18	1.30	18		.72		
Cultural and family values about food and body weight	3.21	1.36	14		.68		
Inconsistent health advice and information	3.05	1.34	21		.61		
*Biological & psychological vulnerabilities* (Cronbach's *α* = .86)	3.19	1.05	* *	* *	* *	* *	* *
Lack of self-esteem	3.32	1.31	10			.79	
Genetics	3.09	1.24	20			.77	
Depression	3.30	1.34	11			.76	
Slow metabolism	3.01	1.30	22			.69	
Poor self-confidence	3.20	1.29	16			.67	
*Self-control & motivation* (Cronbach's *α* = .77)	4.18	0.81	* *	* *	* *	* *	* *
Laziness	4.12	1.04	2				.84
Lack of will power/self-control	4.31	0.89	1				.81
Lack of motivation	4.06	1.02	3				.72
Eight items excluded from further analyses due to similar loadings on two or more components include:							
Low availability of healthy snack food options (e.g. fruits, vegetables)		Lack of family/social support					
A modern lifestyle limits the opportunity for physical activity throughout the day		Maintaining a healthy body weight is not an immediate priority					
Unrealistic expectations to body weight – wanting to achieve a ‘perfect’ body		Physical disability, injury or illness					
Dislike of gyms/exercising		Limited access to healthy foods/exercise facilities					

Note: This table also shows the reliability estimates for the four components.

Significant differences in endorsement level were found between these four barrier components (*F*(2.71, 941.09) = 146.86, *p* < .0001, *η* ^2^ = .30).^3^ The *self-control & motivation* component was the most endorsed by the participants, with a mean rating above the midpoint of the scale. The level of endorsement for this component was significantly higher than that for the other three components (all *p* < .0001). The second most endorsed component was *limited resources/access* which differed significantly from the *nutritional knowledge* and the *biological & psychological vulnerabilities* components (all *p* < .005). No significant difference was observed between the remaining two components of *nutritional knowledge* component and the *biological & psychological vulnerabilities*.

#### Analyses to examine whether beliefs predict participants' actual body mass

Three separate hierarchical multiple regression analyses were conducted to examine whether beliefs about weight gain (i.e. cause, prevention strategies, and barriers) predicted the participants' actual BMI. Dummy coding variables were created for the categorical variables of education, SES, and geographical location to allow for these variables to be entered into the multiple regression analyses. The hierarchical multiple regressions were run with the control measures (i.e. age, gender, education, SES, and geographical location) entered in the first and second steps and the belief components in the final step. This allowed the current researchers to examine whether the belief components predicted significant variance in BMI above and beyond the control variables.

To ensure that multicollinearity was not a problem in the data set, Spearman correlations coefficients were calculated between all predictor variables to identify coefficients above .70. This criterion is recommended by Cohen, Cohen, West, and Aiken (2003) as a cut-off for identifying correlations that are sufficiently high to affect the multiple regression analyses. None of the correlation coefficients met this inter-correlation criterion. As recommended by Field (2009), for each multiple regression the variance inflation factor (VIF) and the tolerance statistic were examined to check for significant multicollinearity in the data. The diagnostic statistics obtained indicated that multicollinearity was not a serious problem in the three sets of analyses (i.e. all VIF values were well below 10, all the tolerance statistics were above 0.2).

The regression examining whether the five causal belief components significantly predicted the participants' BMI scores, after controlling for demographic characteristics of the participants, was not found to be significant, *F* (10, 314) = .946, *p* > .05. In contrast, the other two regressions, examining beliefs about prevention strategies and beliefs about barriers to weight management were found to be significant, *F* (13, 314) = 1.75, *p* = .05 and *F* (12, 303) = 3.09, *p* < .0001, respectively (see Tables 4 and 5).

**Table 4.** Results of the hierarchical multiple regressions examining whether beliefs about prevention strategies against weight gain predict BMI.

Predictor	Step 1	Step 2	Step 3
Gender (1 = male, 2 = female)	−0.014	−0.004	−0.014
Age	0.136	0.137	0.104
High school education vs vocational training		0.007	0.028
High school education vs university education		−0.011	0.009
Middle SES vs low SES		0.111	0.096
Middle SES vs high SES		−0.055	−0.064
Inner-regional vs major city		0.051	0.081
Inner-regional vs outer-regional/remote		−0.120	−0.104
Belief: access to education/exercise			−0.161**
Belief: healthier eating			0.240**
Belief: physical activity			−0.041
Belief: medication/dietary Supplements			0.015
Belief: reduced serving size			0.030
* R*	.137	.167	.260
* R*^2^	.019	.028	.068
* *Δ*R*^2^	.019	.009	.040
* F*	*F*(2, 325) = 3.12*	*F*(8, 319) = 1.14	*F*(13, 314) = 1.75*

Note: Standardised regression coefficients are presented in the table.

*p = .05.

**p < .05.

**Table 5.** Results of the hierarchical multiple regressions examining whether beliefs about barriers to weight management predict BMI.

Predictor	Step 1	Step 2	Step 3
Gender (1 = male, 2 = female)	−0.026	−0.012	−0.038
Age	0.092	0.093	0.055
High school education vs vocational training		0.008	0.005
High school education vs university education		−0.022	−0.002
Middle SES vs low SES		0.132	0.118
Middle SES vs high SES		−0.046	−0.058
Inner-regional vs major city		0.072	0.084
Inner-regional vs outer-regional/remote		−0.116	−0.092
Belief: limited resource/access			0.252**
Belief: nutritional knowledge			−0.378***
Belief: biological & psychological vulnerability			0.157*
Belief: self-control & motivation			−0.005
* R*	.098	.142	.330
* R*^2^	.010	.020	.109
* *Δ*R*^2^	.010	.011	.089
* F*	*F*(2, 313) = 1.051	*F*(8, 307) = .795	*F*(12, 303) = 3.09***

Notes: Standardised regression coefficients are presented in the table.

*p < .05.

**p < .01.

***p < .0001.

The regression model at Step 2 for beliefs about prevention strategies explained 7% of the variance in BMI scores, with the belief components making a significant contribution of 4% variance (*p* < .05). Both the *access to education/exercise* component (*β* = −.16, *p* < .05) and the *healthier eating* component (*β* = .24, *p* < .01) contributed unique variance to the model.

The final regression model for beliefs about barriers to weight management accounted for 10% of the variance in BMI scores, with the belief components making a significant contribution of 7% variance (*p* < .0001). Three belief components of *limited resource/access* (*β* = .25, *p* < .01), *nutritional knowledge* (*β* = −.38, *p* < .0001) and *biological & psychological vulnerabilities* (*β* = .16, *p* < .05) were found to be significant predictors of participants' BMI scores.

As a further test, each participant was coded on the basis of whether they met the BMI criteria for overweight or obesity and separate binary logistic regressions were conducted for prevention and barriers. After controlling for demographic characteristics, participants who provided stronger endorsement for the access to education/exercise component were found to be less likely to be overweight (Wald = 7.00, *p* < .01), whereas those who provided strong endorsement for the healthier eating component were more likely to be overweight (Wald = 12.74, *p* < .0001). None of the four barrier belief components were found to significantly predict the overweight/obesity status of the participants. Entering all the variables in one step did not change the results for the two binary logistic regressions.

## Discussion

The main aim of the present study was to explore and better understand people's beliefs about weight gain with regards to cause, prevention strategies, and barriers to effective weight management. The results of the current study confirmed a number of findings from previous research (e.g. Harvey et al., 2002; Ogden & Flanagan, 2008; Okonkwo & While, 2010). For example, similar to the studies on obesity, the current study found that the foremost causal belief for weight gain was of the individual being responsible for their weight. In other words, weight gain is largely caused by eating too much, not exercising enough, being lazy and lacking in self-control. Consistency between the results of this study and those reported for obesity was also observed with regards to prevention strategies and barriers to weight management. The weight prevention strategies that received the strongest endorsement were those that primarily required the individual to make changes to their lifestyle with regard to their diet and level of physical activity (i.e. strategies contained within the *physical activity* and *healthier eating* components). This is not unexpected given that the majority of these participants regarded weight gain to be primarily the result of a lack of self-control which contributed to the individual eating too much and not exercising enough. As in previous research, the current study found lower levels of support for interventions involving policy change, particularly those that appear to limit individual choice. This was evident in the lower level of endorsement for the *access to education/exercise* component which included community-level interventions (e.g. increasing knowledge of nutrition through education; increasing affordability/accessibility of exercise facilities and relevant professionals through subsidies and local government programmes), and the *reduced serving size* component (i.e. strategies aimed at reducing the portion sizes of convenience foods). The attitude that individuals are largely responsible for their weight also extended to beliefs about barriers to effective weight management. This study found that lack of self-control and motivation were regarded by the lay participants as the most important obstacle to a person effectively managing their weight. However, sound support for the *limited resource/access* component suggests that the participants were also aware that effective weight management can be impeded by difficulties in financing or accessing weight-loss and/or health-related services.

Relative to previous research, the current study obtained a more detailed description of other beliefs that the general public have about weight gain. In addition to beliefs levelled at the individual, participants also acknowledged that weight gain can be caused by social and environmental changes that impact on people's lifestyles (i.e. causes contained within the belief components of *modern living* and *lifestyle limitations*), as well as individual level causes that are outside of the individual's control (i.e. *psychological* and *biological/medical* components). The general public's awareness of the multifactorial causes of weight gain was also evident in the nature of the beliefs components identified in this study for barriers against effective weight management. Participants regarded additional barriers to include poor nutritional knowledge (resulting from a lack of nutritional education, inconsistent health advice or information, and cultural values); as well as biological & psychological vulnerabilities (e.g. genetics, slow metabolism, poor self-esteem, depression). The range and nature of the belief components identified in the current study suggest that lay individuals have a good understanding of the interplay between internal and external factors that not only impact on weight gain, but also prevent successful weight management. This is contrary to the conclusions reached by Jackson, Ball, and Crawford (2001), who argued on the basis of their results that strategies were urgently needed to better educate the general public about the various factors that contribute to weight gain. There are a number of possible reasons for the different findings obtained by these two studies. First, the methodology used in the current study allowed for a more detailed exploration of the various beliefs held by the general community surrounding weight gain, in comparison to the one used in Jackson, Ball, and Crawford's study. Therefore, it could be argued that the current study was able to identify additional beliefs that had not been explored by this previous study. Second, while both studies were conducted on the Australian population, there is approximately a 12-year gap between the two studies. During this period, a number of government-initiated educational campaigns for the prevention of obesity have been implemented via the mass-media (e.g. ‘Measure-Up’ and ‘Go for 2&5’) which would contribute to increasing the general public's understanding about obesity and weight gain.

The degree of consistency between the beliefs held about weight gain to those about obesity suggests that despite obesity being represented as a medical condition, the general public do not differentiate it from weight gain with regards to causes, prevention, and barriers. Furthermore, the level of endorsement for the *medication/dietary supplements* component, which fell below the midpoint of the rating scale, suggests that the general community do not regard weight gain as a medical issue and therefore it does not warrant the use of medical interventions or ‘special’ diets.

The current study also identified an area that may need to be addressed in community-focused educational campaigns, which is that of the contributing role of genetics in body-weight regulation and obesity. In recent decades, great advances have been made in understanding the role of genetics in the regulation of food intake and biological predisposition to obesity (Bouchard, 2007; Choquet & Meyre, 2011). However, the much lower endorsement of biological causes and barriers to weight management obtained in this study suggests that this information has not filtered down to the general community.

Causal explanations about weight gain were not found to predict the BMI of the participants. Instead, BMI was predicted by beliefs held about the prevention strategies and the barriers to effective weight management. Furthermore, participants who believed that weight gain can be prevented by greater access to health education and exercise were less likely to be overweight, whereas those who believed in healthier eating strategies were more likely to be overweight or obese. With regards to the beliefs about barriers to weight management, the components of *limited resource/access*, *biological & psychological vulnerabilities* and *nutritional knowledge* were found to significantly predict the BMI but not the overweight/obesity status of the participants. These results appear to be inconsistent with those obtained by McFerran and Mukhopadyay, who reported that individuals who believed that obesity was caused by a lack of exercise were more likely to be overweight compared to those who believed that obesity was caused by poor diet. They suggested that individuals who believed that lack of exercise, as opposed to other factors, causes obesity are less concerned with regulating their caloric intake and are therefore more likely to have higher BMIs. There are a number of possible reasons for the contradictory findings of the two studies. First, in its exploration of causal explanations of weight gain, the current study did not find a ‘dietary’ dimension or an ‘exercise’ dimension. Instead, items related to dieting/eating practices and exercise were found to load on the same component along with other items related to a lack of self-control and laziness. This component (i.e. *self-control*) was the most strongly endorsed causal dimension of weight gain. In addition, the majority of participants also believed that lack of *self-control and motivation* was the most important barrier to effective weight management. Therefore, these results suggest that participants believe that weight gain is primarily caused by a lack of self-discipline resulting in individuals adopting poor dietary habits and not engaging in enough physical activities. The results of this study also indicate that causal explanations held by lay participants are more complex than simply a ‘dietary’ theory vs an ‘exercise’ theory. However, using less clear-cut causal belief dimensions in the multiple regression analyses may have introduced too much variability in the data to allow for accurate predictions of participants' BMI. Second, differences in personal weight history may have contributed to the contradictory findings. A large proportion of the current participants met the criteria for overweight and obesity. Many had also reported gaining weight and despite actively trying to manage their weight have not been able to return to their usual body weight. While these participants may recognise the importance of healthier eating in the prevention of weight gain, they may not necessarily adopt this dietary practice, which may partly explain their reported difficulty in managing their weight. This finding is consistent with the results of a recent evaluation study conducted by King, Grunseit, O'Hara, and Beuman (2013) of a health education campaign implemented in Australia. They reported that while the campaign was successful in increasing public awareness and understanding about the link between overweight and chronic disease, there were no significant changes in the self-reported consumption of fruit and vegetables or in the level of physical activity undertaken amongst the adults surveyed. Furthermore, Teixeira et al. (2004) reported that in their study on the pre-treatment predictors of attrition and success in a weight-loss intervention programme, a history of dieting predicted unsuccessful future weight-loss attempts. Individuals who have struggled with weight management may have a good understanding of the causes, prevention strategies and barriers associated with weight gain. However, these beliefs may not necessarily be enough to make them adopt long-standing lifestyle changes that result in weight loss.

A limitation of the current study was that the weight gain belief components explained only a small amount of the variance in participants' BMI scores. This finding was not unexpected given that many factors contribute to a person's weight, including environment, family history and genetics, metabolism, as well as behaviour and habits. Of these factors, one would expect for beliefs about weight gain to primarily influence the individual's behaviour and habits. The findings of this study highlight the need for more research into how beliefs/attitudes about weight gain impact on the dieting and exercise behaviours; and the other factors that may mediate this relationship. Future research can also extend on the findings of this study by examining which facets of the *Access to Education/Exercise* and *Healthier Eating* components are best predictive of the participants' BMI scores.

Several other limitations of the current study should be noted. This study was based on self-report, possibly tapping into a social desirability bias. In other words, social desirability bias may have resulted in some participants under-reporting their current weight required for BMI calculations; and over-endorsing dieting/exercise behaviours that are perceived as ‘good’ or ‘health’ behaviours. However, the anonymous nature of the questionnaire should have assisted in reducing this bias. Other limitations included the unequal group sizes, particularly for gender and geographical location. While the study had a relatively large sample size, this sample was skewed to female participation which may have contributed to the non-significant findings obtained for gender in the multiple regression analyses. All effort was made by the current researchers to recruit male participants into the study which included specifically targeting males in shopping centres. However, very few men expressed an interest in participating in this study. The current sample also primarily consisted of individuals living in inner-regional areas of Australia. While this is generally an understudied section of the population, the findings of this study may not be truly representative of the beliefs held by those living in remote geographical areas or those living in a major city. However, it should be noted that the results of the current study are relatively consistent with those obtained by Okonkwo and While (2010), whose sample consisted of undergraduate and postgraduate University students in the UK. Finally, the current study was primarily an exploratory and descriptive study. Hence, replication of the belief dimensions obtained in the current study is needed to confirm that the belief structure identified is representative of the community as a whole.

In conclusion, the findings of this study suggest that community-level interventions that emphasise the individual's responsibility for their weight management (through lifestyle and dietary changes) are most likely to be accepted by the general public. In contrast, intervention strategies that emphasise the use of medicine and dietary supplements, or those that appear to limit individual choice and remove the responsibility of weight management from the individual, are unlikely to receive widespread public support. The findings of this study also suggest a need to better educate the general public about the contributing role of genetic and biological factors on weight gain. Educating the general public of these factors, which are outside the individual's control, may lead to greater acceptance of population-level strategies that are not specifically targeted towards those who are already overweight or obese. This is particularly relevant given the consistency between beliefs about weight gain obtained in the current study and those reported in previous studies for obesity.

## Notes

1. As the assumption of sphericity was violated, the Huynh-Feldt correction was used to calculate the *F*-value.

2. Adjusted for equal variances not assumed.

3. As the assumption of homogeneity of variance was violated, the Welch's *F* was used.
